# Comparison of Lower Eyelid Complications Among Surgical Approaches for Orbital and Zygomaticomaxillary Fractures: A Network Meta-Analysis

**DOI:** 10.3390/jcm15051842

**Published:** 2026-02-28

**Authors:** Yu-Yen Chen, Tai-Yuan Chen, Chun-Min Liang, Pesus Chou

**Affiliations:** 1 Department of Ophthalmology, Taichung Veterans General Hospital, Taichung 407219, Taiwan; yuyenchen.phd@gmail.com; 2 Doctoral Program in Translational Medicine, National Chung Hsing University, Taichung 402202, Taiwan; 3 School of Medicine, National Yang Ming Chiao Tung University, Taipei 112304, Taiwan; 4 Program of Biomedical Informatics and Data Sciences, School of Medicine, Johns Hopkins University, Baltimore, MD 21218, USA; 5 School of Medicine, Chung Shan Medical University, Taichung 402306, Taiwan; 6 Department of Post-Baccalaureate Medicine, College of Medicine, National Chung Hsing University, Taichung 40227, Taiwan; s111086013@mail.nchu.edu.tw; 7 School of Medicine, College of Medicine, Taipei Medical University, Taipei 110301, Taiwan; zxc49852@gmail.com; 8 Department of Medical Education, Taichung Veterans General Hospital, Taichung 407219, Taiwan; 9 Institute of Public Health, National Yang Ming Chiao Tung University, Taipei 112304, Taiwan

**Keywords:** ectropion, entropion, scleral show, scar, orbital fracture, zygomaticomaxillary fracture, network meta-analysis

## Abstract

**Background/Objectives**: This network meta-analysis aimed to evaluate and compare the risks of lower eyelid complications—ectropion, entropion, scleral show, and postoperative scarring—associated with four surgical approaches (subciliary, subtarsal, infraorbital, and transconjunctival) for orbital and zygomaticomaxillary fracture repair. **Methods**: A systematic search of PubMed, Embase, and Cochrane databases identified relevant studies published between 1 January 1990 and 10 January 2026. Twenty-seven eligible studies involving 2790 patients were included. Direct pairwise meta-analyses and network meta-analyses were conducted to compare complication risks among the approaches. Sensitivity analyses were performed to assess the influence of individual studies, and inconsistency tests were applied to evaluate model robustness. **Results**: The subciliary approach was associated with the highest risk of ectropion and scleral show. The transconjunctival approach had the lowest risk of ectropion and scarring but the highest risk of entropion. The subtarsal approach had the lowest risk of scleral show, while the infraorbital approach had the highest risk of postoperative scarring. Sensitivity analyses confirmed consistent rankings, and no significant inconsistency was detected. **Conclusions**: This study provides updated, comprehensive evidence to guide the choice of surgical approach for orbital and zygomaticomaxillary fracture repair. Surgeons should balance operative exposure, cosmetic outcomes, and complication risk, and communicate these trade-offs clearly with patients to optimize decision-making.

## 1. Introduction

The common surgical approaches for orbital and zygomaticomaxillary fractures are subciliary, subtarsal, infraorbital, and transconjunctival approaches. Postoperative lower eyelid malposition, including ectropion, entropion, scleral show, and visible scarring, is the most common complication associated with these approaches. The subciliary, subtarsal, and infraorbital approaches involve transcutaneous incisions of the lower eyelid. The subciliary incision is typically placed approximately 2 mm below the lash line; the subtarsal incision lies 5–7 mm inferior to the lid margin; and the infraorbital incision is made along a natural skin crease at the level of the orbital rim. These three techniques provide excellent access to the infraorbital rim and zygomaticomaxillary complex. However, visible skin incisions may lead to unfavorable aesthetic outcomes. Previous studies have shown that the subciliary approach is associated with a higher incidence of postoperative ectropion, whereas the subtarsal and infraorbital approaches may carry a higher risk of hypertrophic scarring, particularly in younger female patients [1,2,3].

The transconjunctival approach has gained increasing popularity due to its concealed incision and lower incidence of ectropion [4,5]. However, it may be associated with an increased risk of entropion, a condition in which eyelashes turn inward and rub against the ocular surface, potentially causing conjunctivitis or corneal ulceration requiring medical or surgical interventions [6,7]. Additionally, the transconjunctival approach may provide limited surgical exposure unless combined with lateral canthotomy and inferior cantholysis. These additional procedures increase the technical complexity and may contribute to eyelid malposition if not performed meticulously [8]. Therefore, the optimal surgical approach for managing orbital and zygomaticomaxillary fractures remains a subject of debate.

Previous studies on postoperative eyelid complications have mainly focused on comparing the subciliary and transconjunctival approaches [9,10,11,12]. Additionally, most studies are retrospective and include fewer than 50 cases per group, which limits the reliability of the results. Zhang et al., AI-Moraissi et al. and Chen et al. conducted traditional meta-analyses of several studies that directly compared eyelid complications between the subciliary and transconjunctival approaches to enhance the statistical power [13,14,15].

Traditional meta-analysis is limited to direct comparisons between two groups. Conversely, network meta-analysis enables the integration of direct and indirect evidence to simultaneously assess multiple treatment options. This method allows for the estimation of relative risks and rankings across four commonly used approaches: subciliary, subtarsal, infraorbital, and transconjunctival. Al-Moraissi et al. performed a network meta-analysis comparing postoperative eyelid complications among these four approaches and reported that the transconjunctival and subtarsal approaches were associated with the lowest complication rates [16]. Despite the emergence of several new clinical studies involving pairwise comparisons of these approaches [17,18,19,20], no additional network meta-analyses have been conducted since the study by Al-Moraissi et al. Therefore, this study aimed to synthesize more recent evidence and perform an updated and comprehensive network meta-analysis comparing the risk of lower eyelid complications among four surgical approaches.

## 2. Materials and Methods

This study was conducted in accordance with the Preferred Reporting Items for Systematic Reviews and Meta-Analyses (PRISMA) extension guidelines for network meta-analysis [21]. This study was registered with PROSPERO (CRD420251089460). The requirement for ethical approval and informed consent was waived.

### 2.1. Search Strategy

A systematic search was conducted in the PubMed, EMBASE, and Cochrane databases from 1 January 1990 to 10 February 2026, using the following MeSH terms: (“Zygomatic Fractures” [MeSH] OR “Orbital Fractures” [MeSH] OR “Facial Bones/injuries” [MeSH]) AND (“Surgical Procedures, Operative” [MeSH] OR “Fracture Fixation” [MeSH] OR “Fracture Fixation, Internal” [MeSH] OR “Open Reduction” [MeSH]). The records were exported to EndNote, and duplicates were removed. After removing duplicates, articles were screened by titles and abstracts, followed by full-text review for eligibility. Additionally, the reference lists were manually searched for relevant articles.

### 2.2. Inclusion and Exclusion Criteria

The PICO framework was applied as follows: P (Population): patients with orbital or zygomaticomaxillary fractures; I (Intervention): surgeries using one of the following approaches: subciliary, subtarsal, infraorbital, or transconjunctival; C (Comparison): the four approaches; and O (Outcome): ectropion, entropion, scleral show, and scarring.

Only peer-reviewed journal articles were included in the analysis. The inclusion criterion was original prospective, retrospective, or cross-sectional clinical research investigating eyelid complications and directly comparing any two of the following surgical approaches for orbital or zygomaticofacial fractures: subciliary, subtarsal, infraorbital, and transconjunctival. Reviews, meta-analyses, or studies with fewer than 10 cases in either group were excluded from the analysis. Articles were independently assessed by two researchers (Y.-Y. Chen and T.-Y. Chen). Any discrepancies were resolved by discussion, and a third researcher (P. Chou) was consulted if no consensus was reached.

### 2.3. Data Extraction

Data, including the first author, year of publication, number/age of participants, and follow-up duration, were collected from each article. Additionally, the percentage of postoperative complications, including ectropion, entropion, scleral show, and cutaneous scar, was recorded.

### 2.4. Quality Assessment

The risk of bias of the included studies was assessed independently by two reviewers (Y.-Y. Chen and T.-Y. Chen), and any discrepancies were resolved through discussion or consultation with a third reviewer (P. Chou). Randomized clinical studies were evaluated using the Cochrane Risk of Bias 2 (RoB 2) tool, which assesses five domains: bias arising from the randomization process, bias due to deviations from intended interventions, bias due to missing outcome data, bias in measurement of the outcome, and bias in selection of the reported result. Each domain was judged as low risk of bias, some concerns, or high risk of bias, and an overall risk-of-bias judgment was assigned according to the RoB 2 guidance. Non-randomized studies were assessed using the Risk Of Bias In Non-randomized Studies of Interventions (ROBINS-I) tool, which evaluates seven domains: bias due to confounding, selection of participants, classification of interventions, deviations from intended interventions, missing data, measurement of outcomes, and selection of the reported result. Each domain was rated as low, moderate, serious, or critical risk of bias, and the overall risk of bias for each study was determined based on the worst domain rating in accordance with the ROBINS-I guidance [22].

### 2.5. Data Synthesis

#### 2.5.1. Network Geometry

Network meta-analysis was conducted using MetaInsight (version 6.4.0; National Institute for Health Research, London, UK), a web-based platform for network meta-analysis that leverages the netmeta package in R software (version 4.5.2; R Foundation for Statistical Computing, Vienna, Austria) for conducting statistical calculations [23]. Network geometry was illustrated using network plots, which visually represent the relationships between various approaches for fractures, facilitating the evaluation of the clinical evidence strength for each comparison [24,25].

#### 2.5.2. Measures of the Treatment Effect

Measures of the treatment effect were evaluated using odds ratios (ORs). For each outcome (ectropion, entropion, scleral show, and cutaneous scar), the results of the pairwise comparison were summarized as ORs with 95% confidence interval (CI) between the two approaches.

#### 2.5.3. Direct Treatment Comparisons

Direct pairwise comparisons were performed using standard, conventional meta-analyses with a random-effects model. These analyses were conducted for treatment comparisons, including at least two studies.

#### 2.5.4. Mixed Comparisons (Network)

Indirect comparisons were performed using a network meta-analysis. Furthermore, the surgical approaches were ranked. The values of both direct and indirect comparisons were presented in matrices. Treatment rankings were generated using P-scores, which represent the mean probability that each intervention is superior to competing treatments based on the network estimates. P-scores are the frequentist analogue of Surface Under the Cumulative Ranking (SUCRA) and were calculated using the MetaInsight software (Cambridge Research Support Unit, University of Leicester, Leicester, UK; https://crsu-metainsight.le.ac.uk/MetaInsight/, accessed on 10 February 2026).

### 2.6. Statistical Inconsistency Assessment

Inconsistencies were statistically evaluated by comparing the results obtained across direct pairwise and indirect analyses. A two-tailed *p* value of less than 0.05 indicated statistical significance.

### 2.7. Sensitivity Analysis Methods

To confirm the robustness of the overall findings, a leave-one-out sensitivity analysis was performed, removing one study at a time to assess its influence on the combined results.

## 3. Results

### 3.1. Study Identification

**Figure 1:**
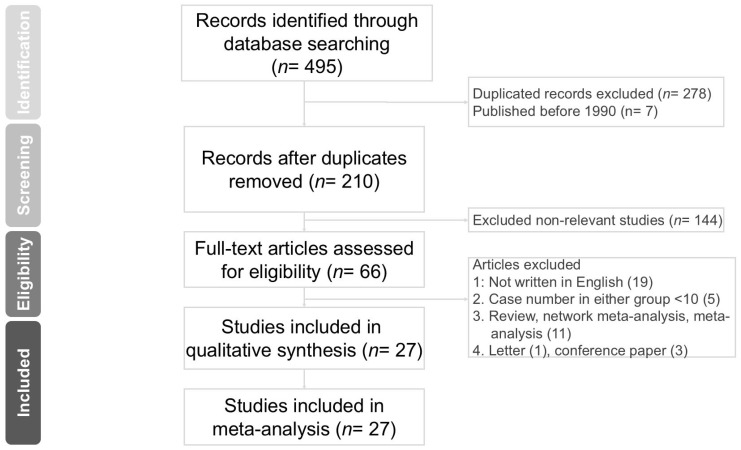
Flow diagram for the study selection process based on the Preferred Reporting Items for Systematic Reviews and Meta-Analyses (PRISMA) guidelines.

The PRISMA NMA extension’s checklist is provided in Table S1. Table S2 shows the search process. A total of 495 articles were retrieved. Figure 1 shows the PRISMA flowchart illustrating the literature screening process. After removing duplicates (*n* = 278), articles published before 1990 (*n* = 7), and non-relevant studies (*n* = 144), 66 studies were screened by reviewing the full texts. Additionally, non-English publications, conference abstracts, reviews, meta-analyses, and studies with fewer than 10 patients in any group were excluded. Finally, 27 studies were included in the analysis [3,4,9,10,11,12,17,18,19,20,26,27,28,29,30,31,32,33,34,35,36,37,38,39,40,41,42].

The 27 studies involved 2790 individuals with orbital, zygomatic, and maxillofacial fractures who underwent surgeries using the following approaches: subciliary, subtarsal, infraorbital, and transconjunctival. Outcomes, including ectropion, entropion, scleral show, and cutaneous scar, were investigated separately. Further details about the first author of each study, the number/age of the participants, and postoperative eyelid complications are shown in Table 1. Most studies were retrospective and compared two approaches. Studies by Mohamed, Ridgeway, Kesselring, Bahr, and Crosara compared three approaches [26,27,32,38,41]. Only one study compared the four approaches [3].

### 3.2. Risk of Bias Assessment

The risk-of-bias assessment is presented in Figure S1. For the randomized controlled trials evaluated using the RoB 2 tool (Figure S1A), most studies were judged as having low risk or some concerns across the assessed domains, with the main source of concern arising from the lack of blinding and deviations from intended interventions, which are common in surgical trials. This resulted in an overall judgment of “some concerns” for the majority of randomized studies. Only one study was rated as having a high risk of bias. For the non-randomized studies assessed using the ROBINS-I tool (Figure S1B), most studies were rated as having low to moderate risk of bias across most domains. However, several studies were judged to have a serious or critical risk of bias, primarily driven by confounding and selection of participants. Overall, the majority of non-randomized studies were considered to have a moderate risk of bias.

### 3.3. Summary of Network Geometry

**Figure 2:**
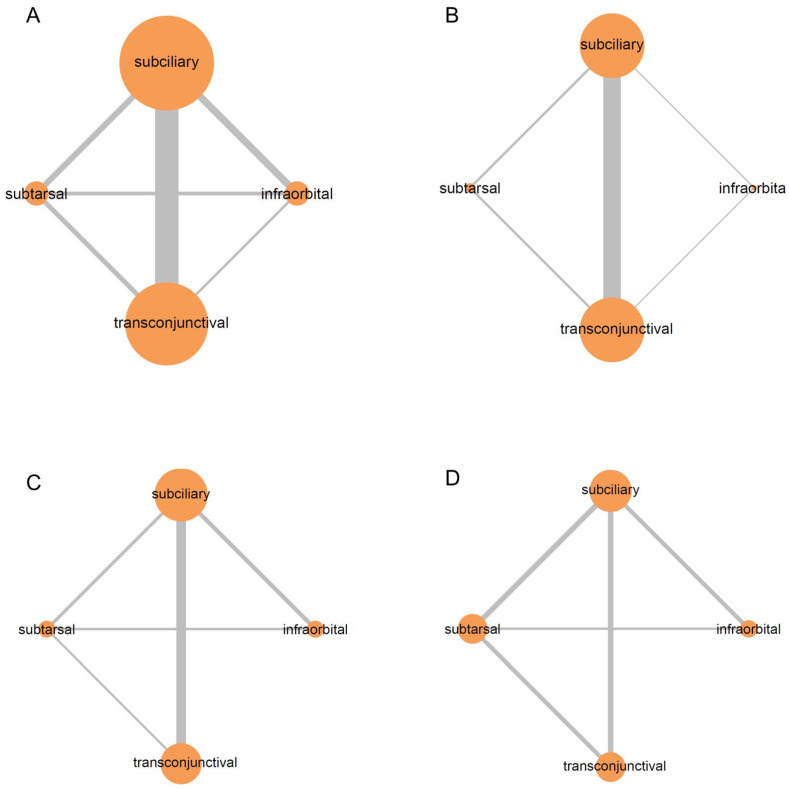
Network plots illustrate the comparisons between various surgical approaches on the postoperative eyelid complications: (**A**) ectropion, (**B**) entropion, (**C**) scleral show, and (**D**) scar.

Figure 2 shows the network plots among the four approaches according to eyelid complications. The size of each node and the thickness of each line represent the number of studies included in the analysis. A large number of studies compared the subciliary and transconjunctival approaches.

### 3.4. Summary of Direct Comparisons of Postoperative Eyelid Complications

#### 3.4.1. Ectropion

Figure S2 shows the pooled results of the meta-analyses for direct comparison of the risk of postoperative ectropion between any two approaches. The transconjunctival approach was associated with a significantly lower risk of postoperative ectropion than the subciliary approach (OR = 0.34; 95% CI: 0.19–0.61). The transconjunctival approach was associated with a lower risk of ectropion than the subtarsal and infraorbital approaches; however, the differences were not statistically significant. The subciliary approach was associated with a higher risk of ectropion than the subtarsal approach; however, the difference was not statistically significant. The risk of postoperative ectropion was similar between the infraorbital, subciliary, and subtarsal approaches.

#### 3.4.2. Entropion

Figure S3 shows the pooled results of the meta-analyses for direct comparison of the risk of postoperative entropion between any two approaches. The transconjunctival approach was associated with a significantly higher risk of postoperative entropion than the subciliary approach (OR = 5.05; 95% CI: 2.12–12.03). The transconjunctival approach was associated with a higher risk of ectropion than the subtarsal approach (OR = 8.62; 95% CI: 0.99–74.90). However, the difference was not statistically significant. The risk of postoperative entropion was similar between the transconjunctival and infraorbital approaches. The subciliary approach was associated with a lower risk of entropion than the infraorbital approach (OR = 1.01; 95% CI: 0.04–25.61). However, the difference was not statistically significant.

#### 3.4.3. Scleral Show

Figure S4 presents the pooled results of the meta-analyses for direct comparisons of the risk of postoperative scleral show between any two approaches. The transconjunctival approach was associated with a significantly lower risk of postoperative scleral show than the subciliary approach (OR = 0.41; 95% CI: 0.20–0.82) but was similar to the subtarsal approach (OR = 0.91; 95% CI: 0.12–7.08). The subciliary approach was significantly associated with a higher risk of postoperative scleral show than the subtarsal approaches (OR = 3.96; 95% CI: 1.36–11.54). The infraorbital approach was associated with a lower risk than the subciliary approach and a higher risk than the subtarsal approach. However, the differences were not statistically significant.

#### 3.4.4. Scar

Figure S5 shows the pooled results of the meta-analyses for direct comparisons of the risk of postoperative scarring between any two approaches. The transconjunctival approach was associated with a significantly lower risk of postoperative scar than the subciliary (OR = 0.18; 95% CI: 0.05–0.61) and subtarsal (OR = 0.16; 95% CI: 0.04–0.73) approaches. The risk of postoperative scar was similar between the subciliary and subtarsal approaches (OR = 0.88; 95% CI: 0.20–3.92). However, the subciliary approach was associated with a significantly lower risk than the infraorbital approach (OR = 0.05; 95% CI: 0.01–0.28). The subtarsal approach was also associated with a significantly lower risk of scar than the infraorbital approach (OR = 0.14; 95% CI: 0.05–0.43).

### 3.5. Network Meta-Analysis

A total of 27 studies were included in the network meta-analysis of ectropion, entropion, scleral show, and scar. The transconjunctival approach was considered the control group.

#### 3.5.1. Network Meta-Analysis: Entropion

**Figure 3:**
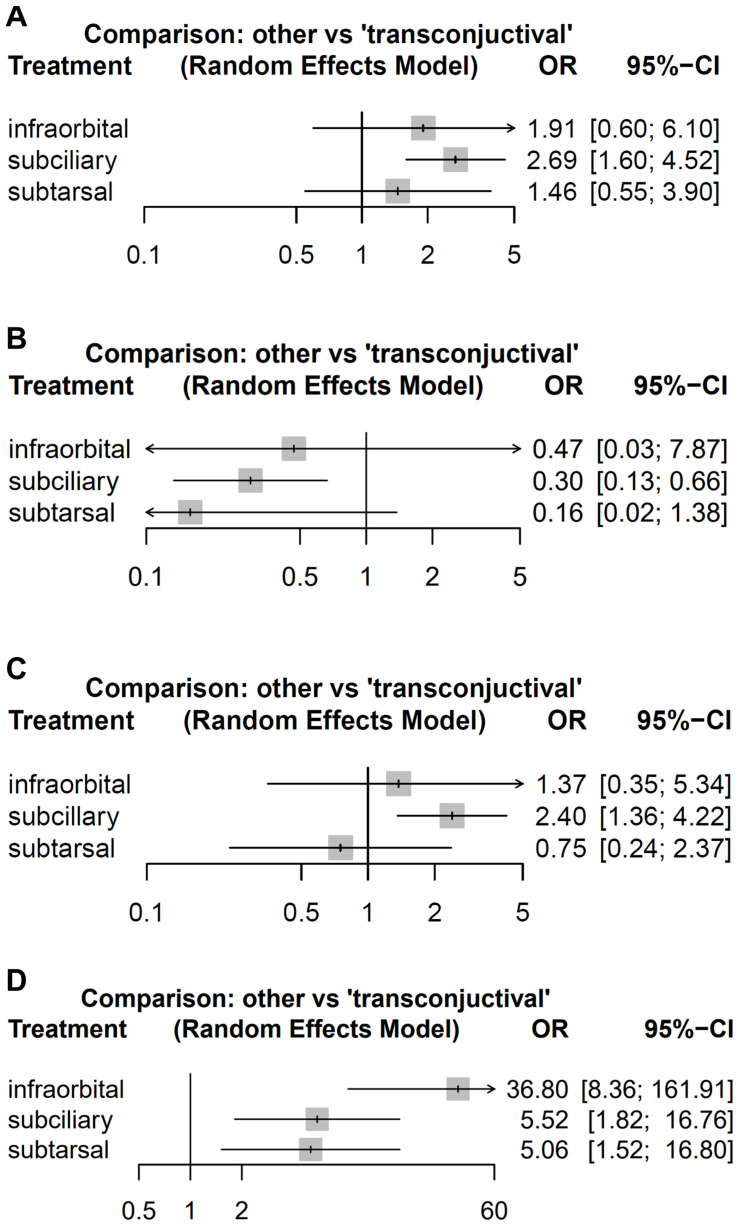
Network Forest plots illustrating the risk of postoperative eyelid complications between different approaches and the transconjunctival approach. Eyelid complications include (**A**) ectropion, (**B**) entropion, (**C**) scleral show, and (**D**) scar.

A total of 2713 patients from 25 studies were included in the comparison of the risk of ectropion. The subciliary group showed a significantly higher risk of ectropion than the control group (OR = 2.69; 95% CI: 1.60–4.52). The risk of ectropion was higher in the infraorbital and subtarsal groups than in the control group. However, the difference was not significant (Figure 3A).

#### 3.5.2. Network Meta-Analysis: Entropion

A total of 1858 patients from 15 studies were included in the comparison of the risk of entropion. The subciliary group showed a significantly lower risk of entropion than the control group (OR = 0.30; 95% CI: 0.13–0.66). The risk of entropion was lower in the infraorbital and subtarsal groups than in the control group. However, no significant differences were observed (Figure 3B).

#### 3.5.3. Network Meta-Analysis: Scleral Show

A total of 1287 patients from 14 studies were included in the comparison of the risk of scleral show. The subciliary group presented a significant increase in the risk of scleral show compared with the control group (OR = 2.40; 95% CI: 1.36–4.22). The risk of scleral show was higher in the infraorbital group and lower in the subtarsal group than in the control group. However, neither reached statistical significance (Figure 3C).

#### 3.5.4. Network Meta-Analysis: Scar

A total of 912 patients from 12 studies were included in the network meta-analysis of the risk of scarring. The subciliary, subtarsal, and infraorbital groups showed a statistically increased risk of scar compared with the control group (Figure 3D).

### 3.6. Rank

The netleague table is a square matrix showing direct and indirect comparisons. The approaches were ranked based on the risks of postoperative eyelid complications.

#### 3.6.1. Rank for Ectropion

The transconjunctival approach had the lowest risk of postoperative ectropion, followed by the subtarsal and infraorbital approaches. The subciliary approach had the highest risk of postoperative ectropion (Table 2).

#### 3.6.2. Rank for Entropion

The subtarsal approach had the lowest risk of postoperative entropion, followed by the subciliary and infraorbital approaches. The transconjunctival approach had the highest risk of postoperative entropion (Table 3).

#### 3.6.3. Rank for Scleral Show

The subciliary approach had the highest risk of postoperative scleral show, followed by the infraorbital and transconjunctival approaches. The subtarsal approach had the lowest risk of postoperative scleral show (Table 4).

#### 3.6.4. Rank for Scar

The transconjunctival approach had the lowest risk of postoperative scarring, followed by the subtarsal and subciliary approaches. The infraorbital approach had the highest risk of postoperative scar (Table 5).

### 3.7. Inconsistency Tests

Inconsistency tests are presented in Table S2 for ectropion, Table S3 for entropion, Table S4 for scleral show, and Table S5 for scar. All *p* values were >0.05, indicating no statistical evidence of a violation of the transitivity assumption.

### 3.8. Results of Sensitivity Analysis

The one-study removal analysis revealed consistent rankings. Therefore, the results of this research were consistent and not influenced by the inclusion or removal of individual studies.

## 4. Discussion

Surgical approaches for orbital and zygomaticomaxillary fractures should be simple, have good access to the targeted area, and have fewer complications. This network meta-analysis investigated the risk of postoperative complications associated with four surgical approaches for orbital and zygomaticomaxillary fractures. The subciliary approach had the highest risk of postoperative ectropion and scleral show. The transconjunctival approach had the lowest risk of postoperative ectropion and scar but had the highest risk of entropion. The subtarsal approach had the lowest risk of scleral show, whereas the infraorbital approach had the highest risk of scar.

Most published articles on orbital and zygomaticomaxillary fractures focus on surgical techniques, and only a few compare the outcomes between surgical approaches. To the best of our knowledge, only one network meta-analysis has been conducted to compare the eyelid complications among the four surgical approaches [16], which showed that the subciliary approach had the highest risk of ectropion, whereas the transconjunctival approach had the highest risk of entropion. These findings are consistent with those of our study. One slight difference is that the previous study showed that the transconjunctival approach had the highest risk of scleral show, whereas our study identified the subciliary approach as having the highest risk. Additionally, our study investigated the risk of postoperative scarring, which was not addressed in the previous analysis.

The subciliary approach may have the highest risk of ectropion because the cutaneous incision is near the eyelid margin, leading to a shortage at the anterior lamella of the eyelid. Therefore, the eyelid margin turns outward. This study showed that the transconjunctival approach had the lowest risk of postoperative ectropion. The transconjunctival approach could avoid skin incision, thereby reducing the risk of postoperative ectropion.

This network meta-analysis revealed that the transconjunctival approach had a higher risk of postoperative entropion compared to the other three approaches. An incision in the conjunctiva could shorten the posterior lamella. Therefore, the eyelid turns inward. Additionally, previous studies have shown that the transconjunctival approach has poor access to the lateral wall unless lateral canthotomy is performed. However, a lateral canthotomy may result in lid malposition [9,12,33,42].

Regarding scleral show, this study revealed that the subciliary approach had a higher risk than the other three approaches. This might be because the subciliary incision passes close to or through the orbicularis oculi muscle and orbital septum—structures essential for maintaining eyelid stability. During postoperative healing, fibrosis and contraction of the deeper tissues can occur, pulling the eyelid downward and resulting in exposure of the lower sclera. Consistent with the findings of the study by AI-Moraissi [16], this study revealed that the subtarsal approach had the least risk of scleral show. This may be due to the anatomical position of the incision and preservation of the eyelid support structures. The subtarsal approach places the incision approximately 4–5 mm below the lower eyelid margin, which is near the natural skin crease and away from the tarsal plate. Therefore, the subtarsal approach minimizes the risk of disrupting critical support structures, such as the orbicularis muscle, lower eyelid retractors, and tarso-ligamentous framework, which are essential for maintaining proper eyelid position. Furthermore, this study revealed that the infraorbital approach had a lower risk of postoperative scleral show than the subciliary approach. The infraorbital approach has the best direct access to the orbital floor compared with other approaches. Therefore, it typically requires less extensive tissue dissection and causes less trauma to the soft tissue, thereby reducing the likelihood of postoperative contracture that pulls the eyelid downward.

The infraorbital approach has the advantage of easy access and good visibility during the operation. However, this study showed that the infraorbital approach was associated with the highest risk of postoperative scar. This finding is consistent with those of previous studies [3,32,38,43]. Unlike the transconjunctival and subtarsal approaches, which place an incision within the conjunctiva to conceal the scar or along natural skin creases, the infraorbital area lacks a skin fold or crease, making any scar noticeable. Additionally, this area is relatively immobile, leading to more prominent scarring. Therefore, this approach is less commonly chosen when aesthetic outcomes are a major concern.

This study has a limitation in that, among the 27 included studies, only 7 were randomized clinical studies. Most of the published studies were retrospective, and potential confounding variables might have affected the choice of surgical approach. In clinical practice, the choice of surgical approach is influenced by factors that may also affect eyelid outcomes, including fracture location and severity, adjunct procedures, variations in surgical technique, timing of surgery, implant and closure methods, patient characteristics, and duration of follow-up. Because most included studies were retrospective, adjustment for these factors was limited, and the assumption of transitivity may not be fully satisfied. In addition, scar outcomes were assessed using heterogeneous measurement scales across studies. Therefore, the findings should be interpreted with caution, and future prospective studies with standardized reporting are warranted. Further prospective, randomized clinical studies are needed. A meta-analysis that includes more randomized studies can result in more objective conclusions.

Another important limitation of this network meta-analysis is the presence of sparse and rare events across several outcomes. Some comparisons were based on a limited number of studies and small event counts, which resulted in wide confidence intervals, particularly for entropion outcomes, and extremely large odds ratios in the scar analysis. These findings suggest that certain estimates may be unstable. Sparse data can also influence the precision of treatment rankings and should, therefore, be interpreted with caution. Future studies with larger sample sizes and more consistently reported outcomes are needed to provide more robust and precise estimates.

This study has several strengths. First, this study involved a large number of patients to provide convincing evidence. Second, direct (traditional meta-analysis) and indirect (network meta-analysis) comparisons were performed. The inconsistency tests revealed no significant inconsistency, indicating that the transitivity assumption held and that the results are persuasive. Third, the sensitivity tests were performed and showed that the removal of any one study could not change our results. The findings of this study highlight the importance of evaluating the potential risk of various eyelid complications before surgery and engaging in communication with patients during the decision-making process.

## Figures and Tables

**Table 1:** Characteristics of the studies included in meta-analysis.

First Author	Year	Study Design	Age	Groups	Num of Pts	Follow-Up	Complications (%)
Bronstein [17]	2020	Retrospective	34.8 ± 12.4	Subciliary	82	at least 6 months	entropion (1.2), ectropion (2.4), corneal injury (7.3), lagophthalmos (1.2), keratoconjunctivitis sicca (3.7)
			35.3 ± 11.9	Transconjunctival	102		entropion (3.9), ectropion (2.0), lagophthalmos (1.0), corneal injury (6.9), keratoconjunctivitis sicca (3.9)
Mohamed [26]	2020	Randomized clinical study	30.9 ± 12.6	Subciliary	15	6 months	entropion (0), ectropion (20), visible scar (13.3), scleral show (26.7), epiphora (13.3)
			37.4 ± 9.0	Transconjunctival	15		entropion (20), ectropion (6.7), visible scar (0), scleral show (13.3), epiphora (20)
			38.3 ± 12.0	Subtarsal	15		entropion (0), ectropion (6.7), visible scar (26.6), scleral show (6.7), epiphora (6.7)
Appling [9]	1993	Retrospective	11–60	Subciliary	25	6 weeks–5 years	scleral show (28.0), ectropion (12.0), canthal malposition (0)
				Transconjunctival	33		scleral show (3.0), ectropion (0), canthal malposition (9.1)
Patel [10]	1998	Retrospective	12–63	Subciliary	30	at least 8 months	entropion (0), ectropion (6.7), visible scar (6.7), scleral show (20)
				Transconjunctival	30		entropion (0), ectropion (0), visible scar (0), scleral show (3.3)
Mehrnoush [18]	2021	Randomized clinical study	34.6 ± 14.2	Subciliary	42	5 months	entropion (0), ectropion (2.4), epiphora (7.1), scleral show (11.9)
			29.0 ± 9.0	Transconjunctival	38		entropion (5.3), ectropion (13.2), epiphora (23.7), scleral show (18.4)
Ridgeway [27]	2009	Retrospective	39	Subciliary	56	6 weeks	entropion (0), ectropion (12.5), lid edema (8.9), scar (3.6),
				Transconjunctival	45		entropion (4.4), ectropion (0), lid edema (0), scar (0)
				Subtarsal	74		ectropion (2.7), entropion (0), scar (1.4), lid edema (1.4)
Trevisiol [11]	2021	Retrospective	44	Subciliary	36	12–74 months	ectropion (8.3), entropion (0)
				Transconjunctival	33		ectropion (0), entropion (0)
Neovius [28]	2017	Retrospective	41	Subciliary	37	at least 6 months	entropion (0), ectropion (8.1), canthal malposition (0), scleral show (11.0)
				Transconjunctival	91		entropion (0), ectropion (2.2), canthal malposition (2.2), scleral show (4.4)
Giraddi [12]	2012	Retrospective	28.4	Subciliary	10	3 months	entropion (0), ectropion (30.0), laceration of tarsal plate (0), button hole laceration of lower eyelid (10.0)
				Transconjunctival	10		entropion (30.0), ectropion (10.0), laceration of tarsal plate (10.0), button hole laceration of lower eyelid (0)
Haghighat [29]	2017	Retrospective	26.7 ± 6.5	Subciliary	17	4 weeks	ectropion (17.6), scar (3.7 ± 0.6) ^a^
				Transconjunctival	17		ectropion (0), scar (0.0 ± 0.0) ^a^
Vaibhav [30]	2016	Randomized clinical study	20–60	Subciliary	20	3 months	entropion (0), ectropion (0), unsatisfactory scar (10)
				Transconjunctival	20		entropion (5), ectropion (0), unsatisfactory scar (0)
Pausch [31]	2016	Retrospective	42.7 ± 21.1	Subciliary	225	6 months	ectropion (3.6), entropion (0), eyelid retraction (0)
				Transconjunctival	121		ectropion (0), entropion (2.5), eyelid retraction (0)
Kesselring [32]	2016	Retrospective	37.5	Subciliary	47	NR	ectropion (2.1), entropion (0)
				Transconjunctival	26		ectropion (0), entropion (0)
				Infraorbital	81		ectropion (2.5), entropion (1.2)
Ishida [4]	2016	Retrospective	NR	Subciliary	29	6 weeks–6.8 years	ectropion (6.9), scleral show (6.9)
				Transconjunctival	179		ectropion (0.6), entropion (3.4), trichiasis (1.1), symblepharon (1.7), lacrimal canaliculus avulsion (1.1), canthal malposition (0.6), conjunctival granulation (2.2)
Salgarelli [33]	2010	Retrospective	37.1	Subciliary	219	6–48 months	ectropion (0), scleral show (1.3), visible scar (17.5)
				Transconjunctival	32		ectropion (0), scleral show (0), visible scar (3)
Raschke [34]	2012	Retrospective	43.3 ± 19.0	Subciliary	114	9 months	ectropion (5.3), scleral show (21.8), entropion (0),
				Transconjunctival	197		ectropion (1.0), scleral show (6.6), entropion (1.0)
Bhatti [35]	2023	Prospective clinical study	32.8	Subciliary	28	5 months	ectropion (14.3), scleral show (11.0), entropion (0), epiphora (21.0)
				Transconjunctival	22		ectropion (13.6), scleral show (17.0), entropion (9.1), epiphora (20.0)
Nowinski [36]	2010	Retrospective	NR	Subciliary	116	3–12 months	ectropion (9.5), entropion (1.7)
				Transconjunctival	40		ectropion (5.0), entropion (2.5)
Subramanian [3]	2009	Randomized clinical study	NR	Subciliary	10	at least 6 months	ectropion (0), scar (1.55) ^b^
				Transconjunctival	10		ectropion (10), scar (1.00) ^b^, prolonged edema (20)
				Subtarsal	10		ectropion (0), scar (1.9) ^b^
				Infraorbital	10		ectropion (20), scar (2.1) ^b^, prolonged edema (20)
EI-Anwar [37]	2017	Randomized clinical study	31.3 ± 9.2	Subciliary	20	6 weeks	ectropion (10), scleral show (15), entropion (0), intolerable pain (10)
			31.6 ± 7.7	Transconjunctival	20		ectropion (0), scleral show (0), entropion (20), intolerable pain (15)
Bähr [38]	1992	Retrospective	9–83	Subciliary	16	6 months–6 years	ectropion (6.3), scleral show (18.8), noticeable scar (0), edema (0)
				Subtarsal	91		ectropion (1.1), scleral show (4.4), noticeable scar (2.2), edema (1.1)
				Infraorbital	23		ectropion (0), scleral show (4.3), noticeable scar (17.4), edema (8.7)
Kumar [19]	2022	Cross-sectional	25.6 ± 3.1	Subciliary	16	3 weeks	firm banding contracture scar (37.5)
			25.6 ± 3.1	Subtarsal	16		firm banding contracture scar (12.5)
Prince [20]	2025	Randomized clinical study	30	Subciliary	11	6 months	ectropion (9.1), scleral show (9.1), scar (0), edema (0), denting (0)
			33.5	Infraorbital	11		ectropion (0), scleral show (0), scar (0), edema (0), denting (0)
Aleem [39]	2017	Randomized clinical study	38.5 ± 8.3	Subciliary	10	at least 6 months	ectropion (0), scleral show (0), noticeable scar (0), lymphedema (0), edema (0)
			29.3 ± 9.5	Infraorbital	10		ectropion (0), scleral show (0), noticeable scar (30.0), lymphedema (0), edema (20.0)
Garvey [40]	2024	Cross-sectional (case series)	48.4	Subtarsal	20	NR	ectropion (25.0), scleral show (10.0), scar (15.0), exposure keratopathy (5.0), trichiasis (0), lid ptosis (0), diplopia (0), lagophthalmos (5.0)
			36.4	Transconjunctival	14		ectropion (7.1), scleral show (0), scar (0), exposure keratopathy (7.1), trichiasis (7.1), lid ptosis (7.1), diplopia (7.1), lagophthalmos (0)
Crosara [41]	2009	Retrospective	31	Subciliary	20	at least 6 months	ectropion (0), scleral show (20), noticeable scar (0), Chronic edema (0)
				Subtarsal	22		ectropion (18.2), scleral show (9.1), noticeable scar (34.1), Chronic edema (0)
				Infraorbital	16		ectropion (6.3), scleral show (18.8), noticeable scar (75.0), Chronic edema (12.5)
Strobel [42]	2016	Retrospective	41.3	Subtarsal	30	6–30 months	scar (23.3), transient epiphora (20), diplopia (6.7)
			45.7	Transconjunctival	15		scar (0), epiphora (33.3), foreign body sensation (6.7), iatrogenic allergic conjunctivitis (6.7)

Pts, patients; NR, not reported; age was presented and mean ± SD, mean, or range; ^a^ VAS score for scar; ^b^ mean scores of scar: 1 for invisible scar, 2 for barely visible scar and 3 points for visible scar.

**Table 2:** Pairwise comparison and ranking of various approaches with respect to ectropion.

Transconjunctival	0.53 (0.13, 2.18)	0.56 (0.08, 4.08)	0.40 (0.23, 0.68)
0.68 (0.26; 1.83)	Subtarsal	1.06 (0.20, 5.47)	0.38 (0.13, 1.11)
0.52 (0.16; 1.67)	0.77 (0.22, 2.63)	Infraorbital	1.19 (0.32, 4.46)
0.37 (0.22; 0.63)	0.54 (0.22, 1.37)	0.71 (0.23; 2.17)	Subciliary

The estimates from the direct pairwise meta-analyses are located above the diagonal line, while the estimates from network meta-analyses are located below the diagonal line. The values represent odds ratios and 95% confidence intervals.

**Table 3:** Pairwise comparison and ranking of various approaches with respect to entropion.

Subtarsal	0.87 [0.05, 14.28]	.	0.14 [0.02, 1.28]
0.53 [0.06, 5.07]	Subciliary	0.86 [0.03, 26.14]	0.30 [0.13, 0.66]
0.34 [0.01, 11.59]	0.63 [0.04, 10.64]	Infraorbital	0.64 [0.02, 19.56]
0.16 [0.02, 1.38]	0.30 [0.13, 0.66]	0.47 [0.03, 7.87]	Transconjunctival

The estimates from the direct pairwise meta-analyses are located above the diagonal line, while the estimates from network meta-analyses are located below the diagonal line. The values represent odds ratios and 95% confidence intervals.

**Table 4:** Pairwise comparison and ranking of various approaches with respect to scleral show.

Subtarsal	0.96 [0.13; 7.40]	0.62 [0.13, 2.92]	0.25 [0.08, 0.80]
0.75 [0.24, 2.37]	Transconjunctival	.	0.43 [0.24, 0.76]
0.55 [0.14, 2.14]	0.73 [0.19, 2.83]	Infraorbital	0.56 [0.16, 2.03]
0.31 [0.11, 0.90]	0.42 [0.24, 0.73]	0.57 [0.16, 2.00]	Subciliary

The estimates from the direct pairwise meta-analyses are located above the diagonal line, while the estimates from network meta-analyses are located below the diagonal line. The values represent odds ratios and 95% confidence intervals.

**Table 5:** Pairwise comparison and ranking of various approaches with respect to scar.

Transconjunctival	0.19 (0.04, 0.90)	0.21 (0.06, 0.72)	.
0.20 (0.06, 0.66)	Subtarsal	0.94 (0.34, 2.58)	0.14 (0.05, 0.43)
0.18 (0.06, 0.55)	0.92 (0.37, 2.25)	Subciliary	0.09 (0.02, 0.43)
0.03 (0.01, 0.12)	0.14 (0.05, 0.38)	0.15 (0.05, 0.49)	Infraorbital

The estimates from the direct pairwise meta-analyses are located above the diagonal line, while the estimates from network meta-analyses are located below the diagonal line. The values represent odds ratios and 95% confidence intervals.

## Data Availability

Data analyzed in this study were a re-analysis of existing data openly available in works cited in the reference section.

## Supplementary Materials

The following supporting information can be downloaded at https://www.mdpi.com/article/10.3390/jcm15051842/s1. Table S1: PRISMA for network meta-analysis checklist; Table S2: Inconsistency test results of the odds ratio in postoperative ectropion for various surgical approaches; Table S3: Inconsistency test results of the odds ratio in postoperative entropion for various surgical approaches; Table S4: Inconsistency test results of the odds ratio in postoperative scleral show for various surgical approaches; Table S5: Inconsistency test results of the odds ratio in postoperative scar for various surgical approaches; Figure S1: Risk of bias of included studies; Figure S2: Pairwise ectropion; Figure S3: Pairwise entropion; Figure S4: Pairwise scleral show; Figure S5: Pairwise scar.

## Abbreviations

The following abbreviations are used in this manuscript:

PRISMA	Preferred Reporting Items for Systematic Reviews and Meta-Analyses
RoB 2	Risk of Bias 2
ROBINS-1	Risk Of Bias In Non-randomized Studies of Interventions
ORs	Odds Ratios
Cis	Confidence Intervals
