# Supplementary material for: Comparison of Lower Eyelid Complications Among Surgical Approaches for Orbital and Zygomaticomaxillary Fractures: A Network Meta-Analysis
Source: J Clin Med. 2026 Feb 28;15(5):1842. doi: 10.3390/jcm15051842 (PMC12986260; doi:10.3390/jcm15051842)
Supplement: Supplementary file 1 [file jcm-15-01842-s001.zip › Table S2 inconsistent test of ectropion.pdf]

Table S2. Inconsistency test results of the odds ratio in postoperative ectropion for various surgical approaches

Comparison	Studies	NMA	Direct	Indirect	Diff	95CIL	95CIU	P Value
Infraorbital : subciliary	6	-0.34	0.18	-1.64	1.82	-0.65	4.29	0.15
Infraorbital : subtarsal	3	0.27	-0.06	0.69	-0.74	-3.23	1.75	0.56
Infraorbital : transconjuctival	2	0.65	0.59	0.68	-0.09	-2.54	2.36	0.94
Subciliary : subtarsal	5	0.61	0.97	-0.39	1.37	-0.72	3.46	0.20
Subciliary : transconjuctival	20	0.99	0.92	2.30	-1.38	-3.80	1.05	0.27
Subtarsal : transconjuctival	4	0.38	0.63	0.14	0.49	-1.47	2.45	0.62

NMA: network meta-analysis; Diff: difference; 95CIL: lower limit of 95% confidence interval; 95CIU: upper limit of 95% confidence interval.
