# Supplementary material for: Comparison of Lower Eyelid Complications Among Surgical Approaches for Orbital and Zygomaticomaxillary Fractures: A Network Meta-Analysis
Source: J Clin Med. 2026 Feb 28;15(5):1842. doi: 10.3390/jcm15051842 (PMC12986260; doi:10.3390/jcm15051842)
Supplement: Supplementary file 1 [file jcm-15-01842-s001.zip › Table S3 Inconsistent test of entropion.pdf]

Table S3. Inconsistency test results of the odds ratio in postoperative entropion for various surgical approaches

Comparison	Studies	NMA	Direct	Indirect	Diff	95CIL	95CIU	P value
infraorbital:subciliary	1	0.45	0.15	1.11	-0.96	-7.01	5.10	0.76
infraorbital:subtarsal	0	1.09	NA	1.09	NA	NA	NA	NA
infraorbital:transconjuctival	1	-0.76	-0.45	-1.40	0.95	-5.08	6.99	0.76
subciliary:subtarsal	2	0.63	0.14	1.53	-1.39	-6.11	3.33	0.56
subciliary:transconjuctival	15	-1.21	-1.21	NA	NA	NA	NA	NA
subtarsal:transconjuctival	2	-1.84	-1.95	1.61	-3.56	-16.12	8.99	0.58

NMA: network meta-analysis; Diff: difference; 95CIL: lower limit of 95% confidence interval; 95CIU: upper limit of 95% confidence interval.
