# Supplementary material for: Comparison of Lower Eyelid Complications Among Surgical Approaches for Orbital and Zygomaticomaxillary Fractures: A Network Meta-Analysis
Source: J Clin Med. 2026 Feb 28;15(5):1842. doi: 10.3390/jcm15051842 (PMC12986260; doi:10.3390/jcm15051842)
Supplement: Supplementary file 1 [file jcm-15-01842-s001.zip › Table S4 Inconsistent test of scleral show.pdf]

Table S4. Inconsistency test results of the odds ratio in postoperative scleral show for various surgical approaches

Comparison	Studies	NMA	Direct	Indirect	Diff	95CIL	95CIU	P Value
infraorbital:subcillary	4	-0.56	-0.57	-0.18	-0.39	-6.34	5.56	0.90
infraorbital:subtarsal	2	0.60	0.47	1.09	-0.62	-3.95	2.71	0.71
infraorbital:transconjuctival	0	0.32	NA	0.32	NA	NA	NA	NA
subcillary:subtarsal	3	1.16	1.37	-0.05	1.43	-1.54	4.39	0.35
subcillary:transconjuctival	9	0.87	0.84	1.93	-1.08	-4.44	2.27	0.53
subtarsal:transconjuctival	2	-0.29	-0.04	-0.40	0.37	-2.10	2.84	0.77

NMA: network meta-analysis; Diff: difference; 95CIL: lower limit of 95% confidence interval; 95CIU: upper limit of 95% confidence interval.
