# Supplementary material for: Comparison of Lower Eyelid Complications Among Surgical Approaches for Orbital and Zygomaticomaxillary Fractures: A Network Meta-Analysis
Source: J Clin Med. 2026 Feb 28;15(5):1842. doi: 10.3390/jcm15051842 (PMC12986260; doi:10.3390/jcm15051842)
Supplement: Supplementary file 1 [file jcm-15-01842-s001.zip › Table S5 Inconsistent test of scar.pdf]

Table S5. Inconsistency test results of the odds ratio in postoperative scar for various surgical approaches

Comparison	Studies	NMA	Direct	Indirect	Diff	95CIL	95CIU	P Value
Infraorbital : subciliary	4	1.90	2.45	1.26	1.19	-1.17	3.56	0.32
Infraorbital : subtarsal	2	1.99	1.95	2.16	-0.22	-2.91	2.48	0.88
Infraorbital : transconjuctival	0	3.60	NA	3.61	NA	NA	NA	NA
Subciliary : subtarsal	5	0.09	0.06	0.19	-0.13	-2.33	2.07	0.91
Subciliary : transconjuctival	5	1.71	1.58	2.23	-0.65	-3.41	2.11	0.65
Subtarsal : transconjuctival	4	1.62	1.66	1.56	0.09	-2.36	2.54	0.94

NMA: network meta-analysis; Diff: difference; 95CIL: lower limit of 95% confidence interval; 95CIU: upper limit of 95% confidence interval.
