# Supplementary figures and images for: Comparison of Lower Eyelid Complications Among Surgical Approaches for Orbital and Zygomaticomaxillary Fractures: A Network Meta-Analysis
Source: J Clin Med. 2026 Feb 28;15(5):1842. doi: 10.3390/jcm15051842 (PMC12986260; doi:10.3390/jcm15051842)

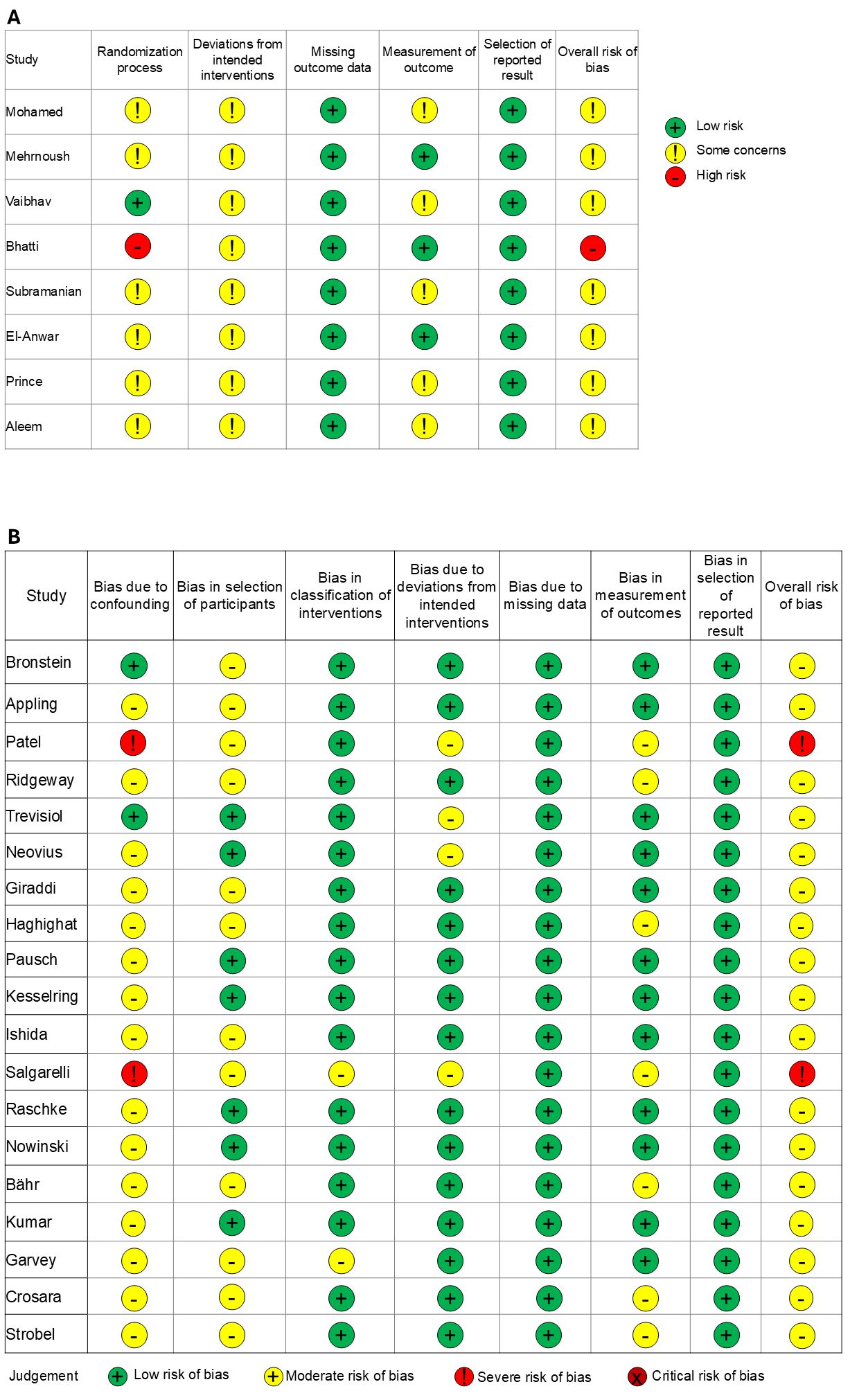

Supplement: Supplementary file 1 [file jcm-15-01842-s001.zip › Figure S1 Risk of bias of included studies.jpg]

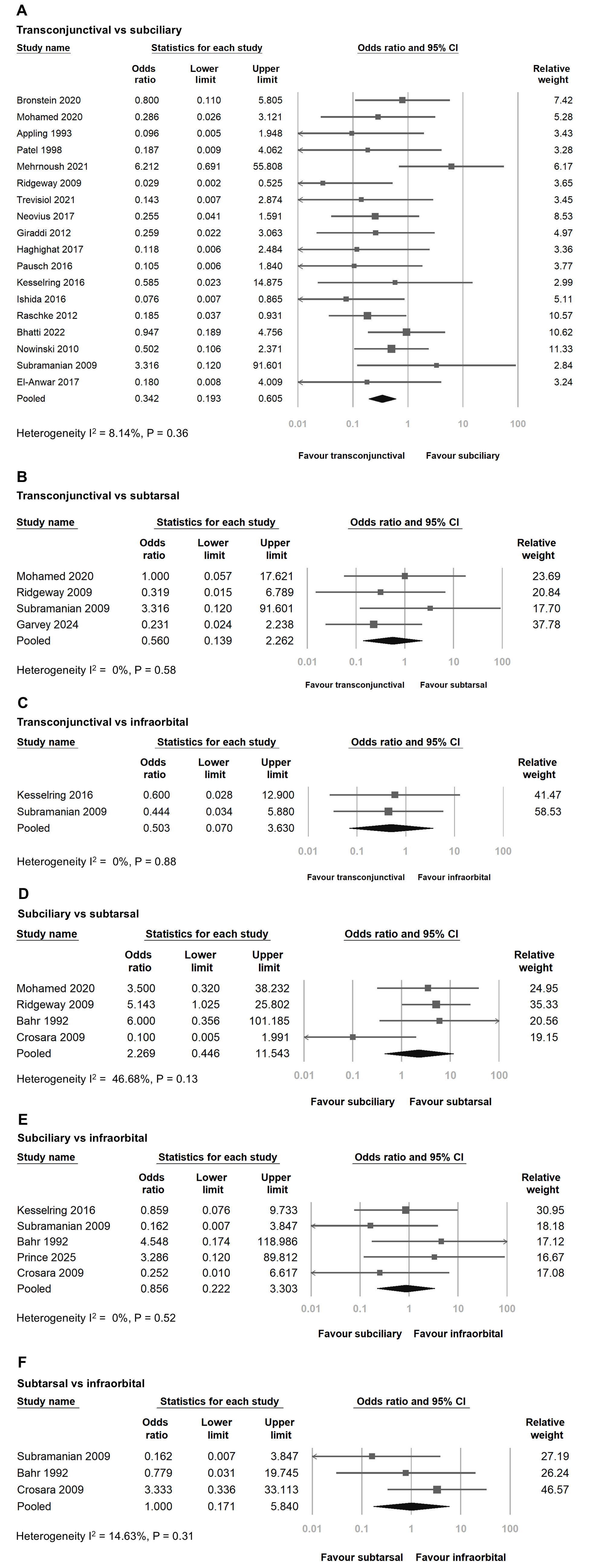

Supplement: Supplementary file 1 [file jcm-15-01842-s001.zip › Figure S2 pairwise ectropion tif.tif]

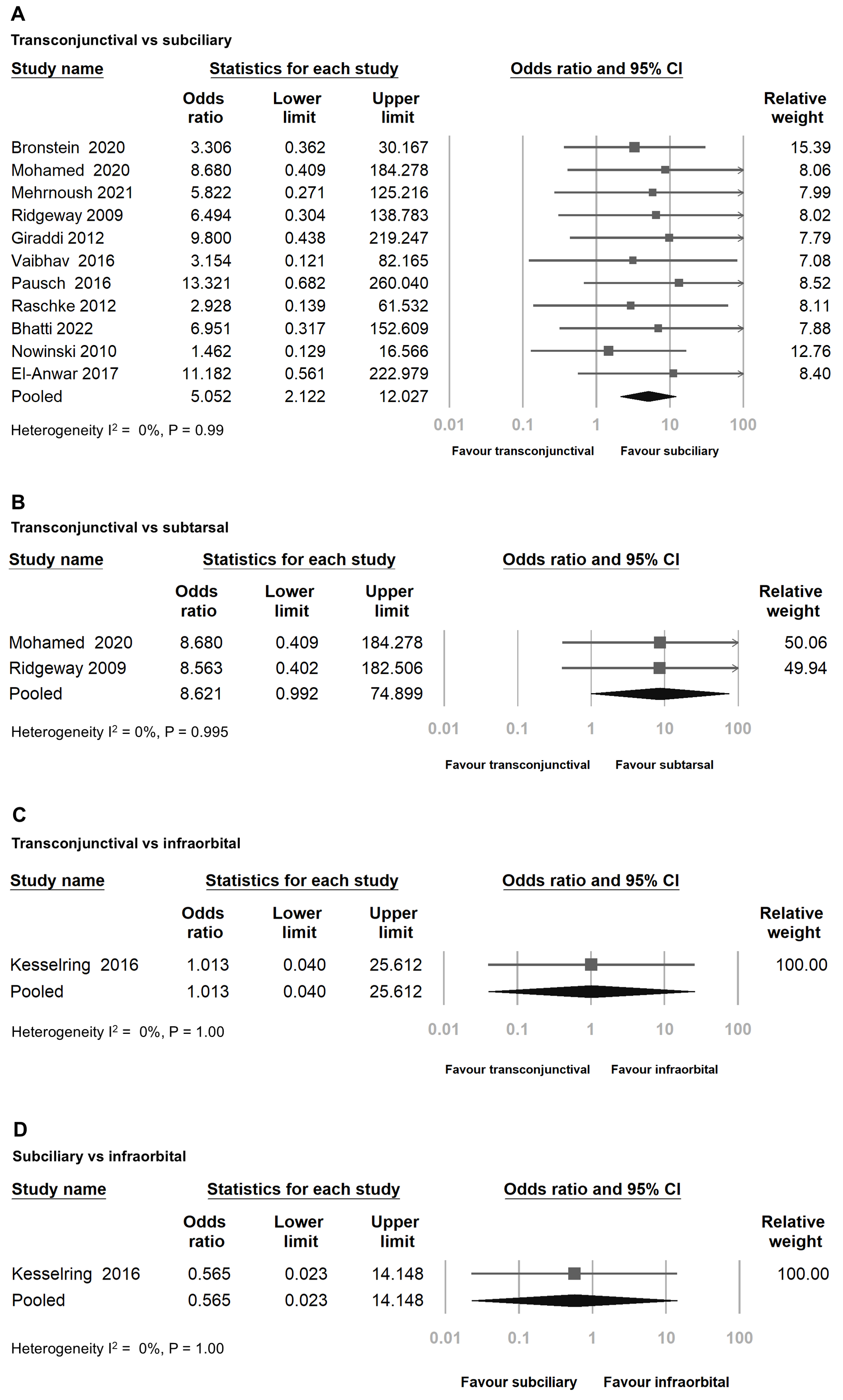

Supplement: Supplementary file 1 [file jcm-15-01842-s001.zip › Figure S3 pairwise entropion tif.tif]

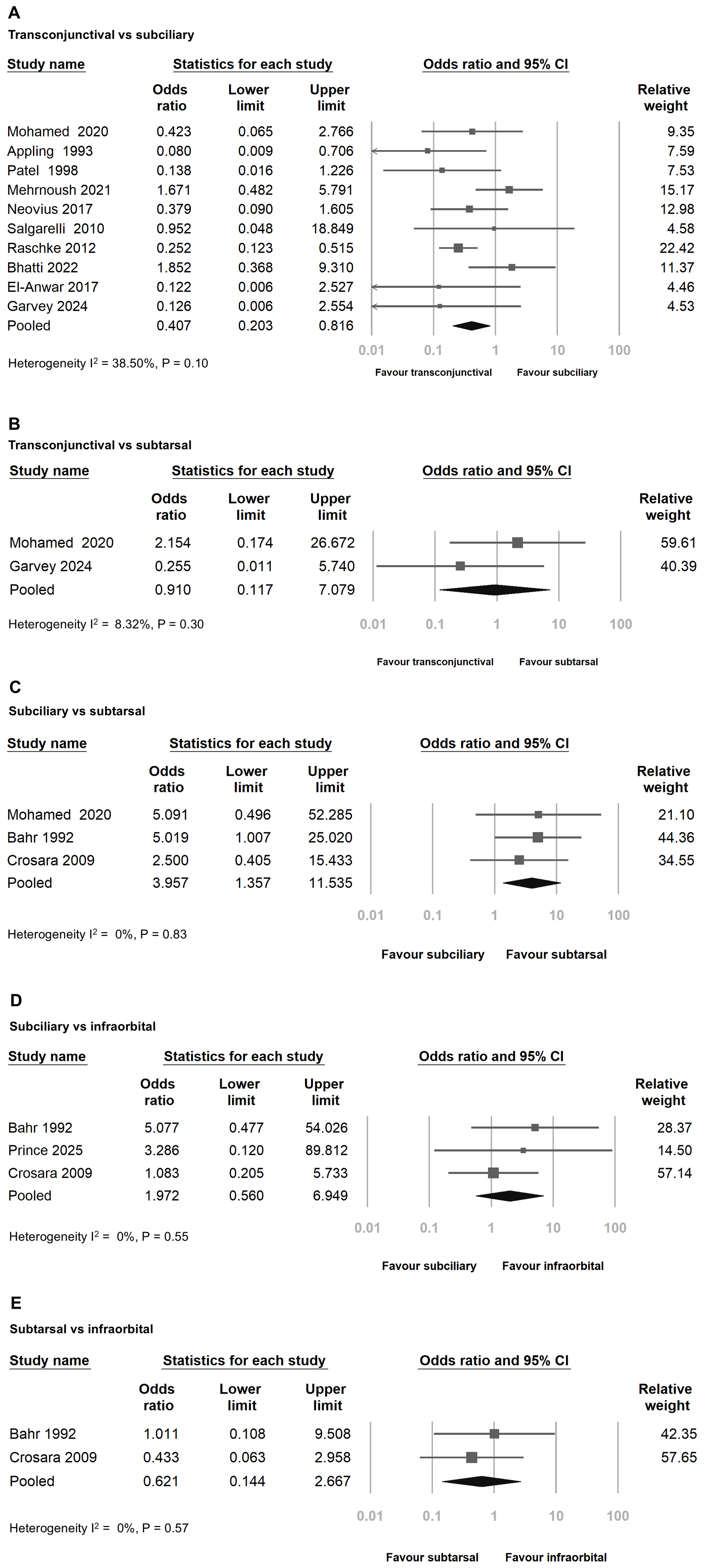

Supplement: Supplementary file 1 [file jcm-15-01842-s001.zip › Figure S4 pairwise scleral tif.tif]

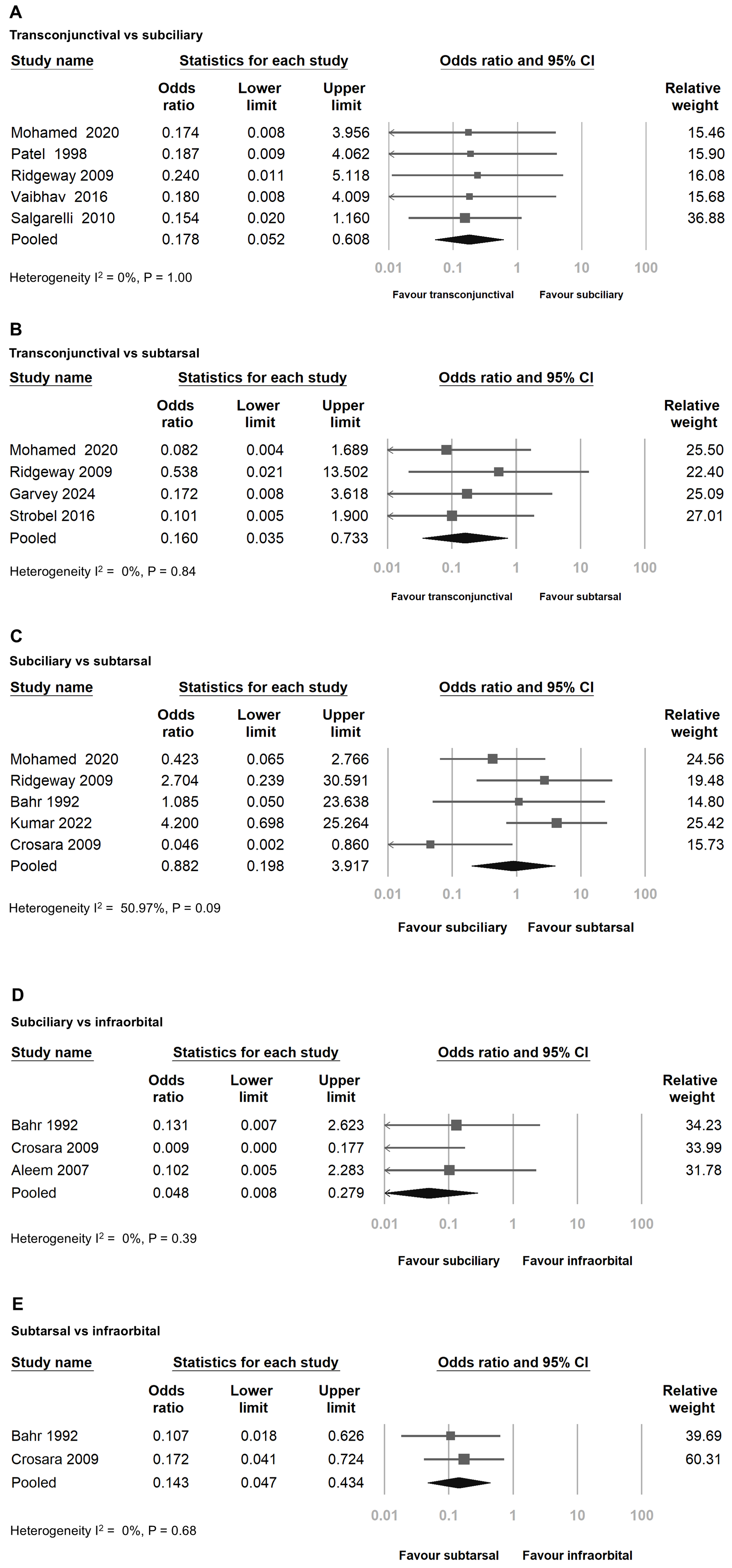

Supplement: Supplementary file 1 [file jcm-15-01842-s001.zip › Figure S5 pairwise scar tif.tif]
